# Osteogenic enhancement of modular ceramic nanocomposites impregnated with human dental pulp stem cells: an approach for bone repair and regenerative medicine

**DOI:** 10.1186/s43141-022-00387-4

**Published:** 2022-08-17

**Authors:** Eman E. A. Mohammed, Hanan H. Beherei, Mohamed El-Zawahry, Abdel Razik H. Farrag, Naglaa Kholoussi, Iman Helwa, Mostafa Mabrouk, Alice K. Abdel Aleem

**Affiliations:** 1 Medical Molecular Genetics Department, Human Genetics and Genome Research Institute, National Research Centre, Cairo, Egypt; 2 Refractoriness, Ceramics and Building Materials Department, Inorganic Chemical Industries and Mineral Resources Research Institute, National Research Centre, Cairo, Egypt; 3 Fixed and Removable Prosthodontics Department, Oral and Dental Research Institute, National Research Centre, Cairo, Egypt; 4 Pathology Department, Medicine and Clinical Studies Research Institute, National Research Centre, Cairo, Egypt; 5 Stem Cell Research Group, Medical Research Center of Excellence, National Research Centre, Cairo, Egypt; 6 Immunogenetics Department, Human Genetics and Genome Research Institute, National Research Centre, National Research Centre, Cairo, Egypt

**Keywords:** Human dental pulp stem cells, hDP-MSCs, Osteogenic differentiation, Ceramic nanocomposites, Bioactivity, Biocompatibility

## Abstract

**Background/aim:**

Human dental pulp-derived mesenchymal stem cells (hDP-MSCs) are a promising source of progenitor cells for bone tissue engineering. Nanocomposites made of calcium phosphate especially hydroxyapatite (HA) offer an impressive solution for orthopedic and dental implants. The combination of hDP-MSCs and ceramic nanocomposites has a promising therapeutic potential in regenerative medicine. Despite the calcium phosphate hydroxyapatite (HA)-based nanocomposites offer a good solution for orthopedic and dental implants, the heavy load-bearing clinical applications require higher mechanical strength, which is not of the HA’ properties that have low mechanical strength. Herein, the outcomes of using fabricated ceramic nanocomposites of hydroxyapatite/titania/calcium silicate mixed at different ratios (C1, C2, and C3) and impregnated with hDP-MSCs both in in vitro cultures and rabbit model of induced tibial bone defect were investigated. Our aim is to find out a new approach that would largely enhance the osteogenic differentiation of hDP-MSCs and has a therapeutic potential in bone regeneration.

**Subjects and methods:**

Human DP-MSCs were isolated from the dental pulp of the third molar and cultured in vitro. Alizarin Red staining was performed at different time points to assess the osteogenic differentiation. Flow cytometer was used to quantify the expression of hDP-MSCs unique surface markers. Rabbits were used as animal models to evaluate the therapeutic potential of osteogenically differentiated hDP-MSCs impregnated with ceramic nanocomposites of hydroxyapatite/tatiana/calcium silicate (C1, C2, and C3). Histopathological examination and scanning electron microscopy (SEM) were performed to evaluate bone healing potential in the rabbit induced tibial defects three weeks post-transplantation.

**Results:**

The hDP-MSCs showed high proliferative and osteogenic potential in vitro culture. Their osteogenic differentiation was accelerated by the ceramic nanocomposites’ scaffold and revealed bone defect’s healing in transplanted rabbit groups compared to control groups. Histopathological and SEM analysis of the transplanted hDP-MSCs/ceramic nanocomposites showed the formation of new bone filling in the defect area 3 weeks post-implantation. Accelerate osseointegration and enhancement of the bone-bonding ability of the prepared nanocomposites were also confirmed by SEM.

**Conclusions:**

The results strongly suggested that ceramic nanocomposites of hydroxyapatite/ titania /calcium silicate (C1, C2, and C3) associated with hDP-MSCs have a therapeutic potential in bone healing in a rabbit model. Hence, the combined osteogenic system presented here is recommended for application in bone tissue engineering and regenerative medicine.

## Background

Human dental pulp stem cells (hDP-MSCs) provide a promising and easily accessible source of mesenchymal stem cells (MSCs) for regenerative medicine. The hDP-MSCs have a high proliferative prospective and expansion rate, self-renewal ability, and multilineage differentiation capability. The hDP-MSCs can differentiate into a variety of cell types, including odontoblastic, adipogenic, and neural cells. This grades DP-MSCs as a promising and attractive source for regenerative medicine and tissue engineering [1–4].

Human DP-MSCs play an important role in repairing bone defects involving the vertebral spine and craniofacial bone defects, which can be caused by injuries, congenital anomalies, trauma, and different diseases [5, 6]. The hDP-MSCs have been used in bone tissue regeneration in combination with 3D nanostructured scaffolds in the induced bone defects of animal models. Experiments performed on calvarias defect model rat showed prospective outcomes in the formation of new bones as examined by histopathology and immunohistochemistry [7].

Mesenchymal stem cells are known to positively express CD44, CD73, CD90, CD105, and CD271, whereas showed a lack of expression of markers CD34, CD45, and HLADR [8, 9]. However, there are no specific exact markers that characterize the DPSC that is considered a heterogeneous population [9]. In fact, different mesenchymal stem cell markers have been used to select different subsets of DPSCs that exhibit different biological behaviors [9, 10].

The in vivo studies using animal models have shown the importance of MSCs transplanted to enhance bone defect healing and subsequently the stiffness of the regenerated structure. In various animal models, MSCs used to repair critical size bone defects. In previous studies, using rabbits as an animal model for bone regeneration, experiments were performed at different bone sites, including the mandible, femur, calvarias, and radius [11, 12]. We have shown that the hDP-MSCs utilized in the healing of the induced bone defect of temporomandibular joint (TMJ) in a rabbit model had a great potential for bone healing [13]. Moreover, the osteogenically differentiated DP-MSCs showed higher bone healing efficiency than the undifferentiated ones at 9 weeks post-transplantation.

Promising platforms for bone tissue engineering are represented by nano biomaterials and nanocomposites, as they mimic the natural extracellular matrix (ECM) and induce bone formation due to their higher surface area and small particle size. In addition, when these nanomaterials are based on calcium phosphate or calcium silicate materials, they will provide a good substrate for bone formation. Several conditions governs the performance of those nanomaterials including porosity, nutrient exchange, and protein adsorption. These conditions involve, but are not limited to the surface area, roughness, wettability, and the other inherent properties of nanocomposites [14–16].

New approach in the inorganic biomaterials implant recently was vastly grown, namely calcium silicate-based ceramics (CS) [17]. Confirmed enhanced osteoblast proliferation and differentiation for both calcium (Ca) and silicon (Si) ions, since they are considered essential elements for the human body. Enhanced bioactivity behavior is to be achieved through the incorporation of those elements within nanocomposite materials [18, 19].

Bone replacement and regeneration applications both in vitro and in vivo were developed using ceramic calcium silicates especially CaSiO_3_ and Ca_2_SiO_4_ phases, due to their capabilities to release Ca and Si ions during the implantation process [20]. However, a major drawback of the CaSiO_3_ ceramics is their high dissolution rate, which leads to increased pH in the media that could be lethal for the cells [21]. Apart from the new technologies of densification, another technique was developed to overcome this limitation by introducing some reinforcing agents such as metal oxides [22]. Reports on titania (TiO_2_) enhanced nanocomposites that are based on silicate bioactive glass were described [22, 23].

In this context, we hypothesize that the fabrication of nanocomposites composed of three different ceramics, namely hydroxyapatite, titania, and calcium silicate, will generate a nanocomposite with impressive properties suitable for the replacement of damaged bones. These originally prepared nanocomposites were characterized by X-ray diffraction (XRD) technique, Fourier transform infrared spectroscopy (FTIR), compressive strength, and scanning electron microscopy (SEM) before and after the biological studies.

We investigated the osteogenic potential of hDP-MSCs when combined with ceramic nanocomposites of hydroxyapatite/titania/calcium silicate (C1, C2 and C3) in vitro experiments. In vivo experiments were also performed to evaluate the therapeutic potential of the present approach through transplantation in induced defects of the rabbit’s tibia.

## Subjects and methods

### Subjects

The dental pulps were extracted at the dental clinic of the National Research Centre in Egypt. Third molars were collected from four participants aged 25–35 years. Human dental pulp of third molar teeth was collected from healthy participants, who had neither infected nor carious teeth, and was used in the current study. The teeth were partially impacted with simple extraction without any traumatic injury to the tooth structures, leading to pulp exposure, and without being contaminated; this simple extraction preserves the sterile pulp. Another resource to get sterile dental plate pulp was the extraction of premolar teeth for orthodontic treatment to obtain more space in the dental arch for the future movements of all teeth in the right way.

### Experimental animals

In this study, a total of 18, 6-month-old New Zealand white rabbits (1.5–2 kg) were used. All animals were treated humanely under the National Research Centre Ethics Approval Committee. Rabbits were obtained and caged at cool room temperature away from direct sunlight to protect them from draughts, loud noises, and direct access to the National Research Centre Animal House radiators. Rabbits were fed the available commercial pelleted rabbit diet.

### Experiment design

Rabbits were sacrificed at 4 weeks post-transplantation and classified into 3 experimental groups (G1, G2, G3); each group included 3 rabbits; the tibia defect has been transplanted with the osteogenically differentiated hDP-MSCs combined with the different types of ceramic nanocomposite scaffold (C1, C2, C3) in vitro, and 3 control groups (G4, G5, G6), each group included 3 rabbits, the tibia defect received different types of ceramic nanocomposite scaffold (C1, C2, C3) in the absence of osteogenic differentiated hDP-MSCs cells (Table 1).

**Table 1:** Classification of the animal groups

Ceramic nanocomposite scaffolds	Rabbits (experimental)	Rabbits (control)
**C1**	3 (G1)	3 (G4)
**C2**	3 (G2)	3 (G5)
**C3**	3 (G3)	3 (G6)

### Ethical consideration

All participants were informed about the practical steps of this study and signed approval consent. This study has approved by the Ethics Committee of the National Research Centre and followed the ethical approvals of animal protocol (Approval No. 16/263).

## Methods

### In vitro study

#### Isolation of dental pulp-derived mesenchymal stem cells

Dental pulp tissue was digested by the collagenase/dispase enzymatic digestion method [24]. Dental pulps were extracted from healthy third molar teeth by mechanical fracturing and the dentinal excavator that acts gently to extract the dental pulp. The extracted pulp was immersed for an hour at 37 °C in a digestive solution of two enzymes, collagenase type I (3 mg/ml) and dispase (4 mg/ml), and then centrifuged at 1000 rpm/min for 10 min. The cell pellets were collected and cultured in alpha minimal essential medium (α-MEM; Gibco BRL, Life Technologies B.V., Breda, Netherlands, containing 10% fetal bovine serum (FBS; Gibco ERL), 100 U/ml penicillin (Gibco ERL), and 100 U/ ml streptomycin (Gibco ERL) and incubated at 37 °C under standard conditions with 5% CO2. The propagated cells at the third passage were used for the osteogenic differentiation experiment.

#### Culture and proliferation of dental pulp-derived mesenchymal stem cells

Culture and proliferation of hDP-MSCs were maintained in regular proliferation media until a confluency of approximately 80% was reached, and then, cells were passaged and reseeded. Manual scraping techniques were performed using a cell scraper (Corning Incorporated, Costar, Mexico), cell collection was performed, and then centrifugation and resuspension were performed in regular proliferation media, and the cells were reseeded in culture plates [24].

#### Osteogenic differentiation of dental pulp stem cells

At the third passage, the human DP-MSCs proliferation when at 70% confluence has been used for osteogenic differentiation experiment by replacing the regular culture media with osteogenic differentiation media containing DMEM, 20% FBS, L-ascorbic acid 2-phosphate, β-glycerol phosphate, dexamethasone 100 μg/ml penicillin/streptomycin, and 1% glutamax [25]. The control culture plates (MSCs in regular proliferation media) were set for each sample. Two similar sets with different incubation times were simultaneously initiated, One set, the experimental plates in differentiation media and control plates in regular media, was incubated for 14 days prior to proceeding to the characterization protocol, while the second set of the same contents was incubated for 29 days before the characterization.

#### Characterization for osteogenic-differentiated dental pulp stem cells

Characterization of osteogenic differentiation DP-MSCs was performed using Alizarin Red staining to detect mineralized nodules generated in the osteogenic-differentiated cultures. Briefly, the plates were washed 3 times with PBS, fixed in 70% ethanol at room temperature for 1 h, washed with dH_2_O, and then, 1.3% Alizarin Red staining dye (pH 4.2) was added. The plates were incubated at 37 °C for 1 h with gentle shaking. The plates were washed with dH_2_O until disappearing the dye color. The dH_2_O was carefully aspirated, the plates were washed with PBS, and sufficient dH_2_O was added to cover the cell, prepared for inverted microscope imaging [26].

#### Osteogenic differentiation of DP-MSCs on ceramic nanocomposites discs

Osteogenic differentiation of DP-MSCs was achieved in vitro at the prepared ceramic nanocomposite discs (C1, C2, C3) 9.5 mm in diameter and 4 mm in height. Ceramic nanocomposite discs were sterilized by washing twice in a 6-well culture plate with 70% ethanol, exposed to UV radiation for 1 h, were pre-hydrated in phosphate-buffered saline (PBS) for 15 min, and washed twice with the osteogenic medium (1 h for each rinse). The DP-MSCs were seeded on ceramic nanocomposite discs (C1, C2, C3) at a density of 10 × 10^6^ and incubated at 37 °C in the osteogenic differentiation medium, DMEM containing 20% FBS, 100 μg/ml penicillin and streptomycin, and 1% glutamax, L-ascorbic acid 2-phosphate, β-glycerol phosphate, and dexamethasone for 3 weeks; the medium was exchanged every 3 days [25]. The ceramic nanocomposite discs containing osteogenic-differentiated DP-MSCs were fixed with glutaraldehyde on the 21st day and analyzed by scanning electron microscope (SEM).

##### In vivo study

At the third passage (P3), human dental pulp stem cells (hDP-MSC) that were incubated, for 3 weeks, along with the prepared ceramic nanocomposite, in the osteogenic differentiated media in vitro were surgically transplanted into the rabbit tibial defect for four weeks prior to rabbit’s sacrifices.

##### Implantation of DP-MSCs seeded on ceramic nanocomposites into rabbit’s tibia

Preoperatively, each rabbit has fasted for 12 h. General anesthesia has been done through intramuscular injection of ketamine hydrochloride (35 mg/kg BW) and xylazine (5 mg/kg BW) and continued during the surgical procedure. The external incision in the tibia, the skin incisions (about 1.5 cm) had been performed initiated), and extending to the muscles tissue layers deep to the bone level, generating a defect cavity in the tibia 0.5 mm in diameter and 0.5 mm in depth. The cavities had been made using standard spherical bur in a contra-angle hand piece running at approximately10,000 rpm and abundantly irrigated with saline solution. Finally, the flaps had been repositioned cautiously and sutured in layers. The pain after the surgical operation was controlled by the injection of buprenorphine (50 μg/kg BW) every 2 h for the first day. This experiment was performed following the national and European guidelines for animal experiments. The hDP-MSCs (about 2 million cells) collected at P3 were seeded at the three prepared ceramic nanocomposites (C1, C2, C3), for 2 days in normal proliferation media and exchanged with the osteogenic differentiated media after PBS washing and incubated for 3 weeks at 37 °C. The scanning electron microscope (SEM) was evaluated to assess the osteogenic differentiation perspective of the osteogenic-differentiated hDP-MSCs cells combined with the ceramic nanocomposite discs (C1, C2, C3). Meanwhile, the combination system of osteogenically differentiated hDP-MSCs cells and ceramic nanocomposite discs (C1, C2, C3) was applied and fitted in the rabbit tibial defect to assess the newly formed bone and bone regeneration potential.

##### Scanning electron microscope

Using scanning electron microscope (SEM), the images of ceramic nanocomposites discs seeded with hDP-MSCs after 3 weeks in vitro cultures were captured. Analysis of SEM images aims to evaluate the combination potential of hDP-MSCs and different types of ceramic nanocomposite discs (C1, C2, C3) on the enhancement of osteogenic differentiation and new bone formation in the rabbit model.

Samples preparation for SEM imaging involved the following: the cells were fixed on the surface of nanocomposite discs using 4% glutaraldehyde and then dehydrated in a graded ethanol solution (15%, 25%, 35%, 45%, 70%, and 95% ethanol) each for 10–20 min. This was followed by attaching samples onto the sample holders and coated with an ultrathin layer of gold in a coating apparatus and then observed by SEM accompanied with energy-dispersive X-ray spectrometry (EDS).

##### Expression of the DP-MSCs surface markers

At the third passage (P3), the expression of certain DP-MSCs’ markers was quantified using the fluorescein-activated cell sorting (FACS) Calibur (BD Biosciences, USA). The DP-MSCs cells were trypsinized (0.25% trypsin and 0.01% EDTA; w/v), washed twice with PBS containing 0.5% bovine serum albumin (BSA; Sigma-Aldrich, Saint Louis, MO, USA), and resuspended in PBS with concentration of 2 × 10^5^ cells/20 μl. Consequently, the tagged cells were incubated in 10 μl of the fluorescently labeled antibodies; CD34-PE, CD73-APC, CD90-FITC, and HLA-DRFITC (BDB Biosciences, USA) in a dark place at room temperature for 30 min. Isotype-matched controls were obtained in each analysis to assess the potential for nonspecific staining and autofluorescence [27].

Flow cytometric analysis was ran, and 10,000 events were collected. Data analysis were performed to detect and calculate the positive percentage for each antibody [27].

## Biomaterial

### Materials and methods

#### Materials

Calcium nitrate tetrahydrate (Ca (NO_3_)_2_.4H_2_O) (*MW* = 236.15 g/mol, Merck) and diammonium hydrogen phosphate NH_4_H_2_PO_4_ (*MW* = 115.03 g/mol, Sigma Aldrich) were used as starting materials to prepare the HA powder. Ammonium hydroxide (NH_4_OH) solution was used to adjust the pH value, while the ethylene glycol (C_2_H_4_(OH)_2_) was used as a dispersant with calcium nitrate tetrahydrate. All these chemical reagents were of analytical grade, provided from Aldrich, and were used without further purification. Tetraethyl orthosilicate (TEOS, 98% Si (OC_2_H_5_)_4_, *MW* = 208.33 g/mol, Aldrich) was used for the preparation of nano-calcium silicate. The used TiO_2_ powder (anatase) for preparation of the nanocomposites was provided in the form (*MW* = 80 g/mol, Sigma).

### Methods

#### Nano-sized HA powder

The coprecipitation method was chosen for the preparation of nano-hydroxyapatite (nHA); ethylene glycol was used as a dispersant medium that would likely generate the desired fine grain powders. In addition, it is expected that it would produce nHA powder with a single phase in crystalline apatite and grain size at low temperatures. Practically, Ca (NO_3_)_2_.4H_2_O and (NH_4_)_2_ HPO_4_ were used as starting calcium and phosphorus precursors. Ammonia [NH_3_, (Merck)] was just used to adjust the pH of the solution. Using a peristaltic pump, 1.67 molar solution of Ca (NO_3_)_2_.4H_2_O dissolved in ethylene glycol (C_2_H_4_ (OH)_2_) (pH = 10) was added at a constant rate of 3 ml/min onto a molar solution of (NH_4_)_2_HPO_4_ under vigorous stirring at room temperature, and Ca/P = 1.67 was maintained. After the white precipitate solution was obtained, it was left to age for 24 h at room temperature. The obtained gel was filtered and washed repeatedly using double-distilled water in order to remove the NH_4_ (aq) and NO_3_ (aq) residuals. The filtered cake obtained by this process was then dried in an oven at 80 °C for 48 h [28].

#### Calcium silicate (Ca SiO3)

The nano-calcium silicate (1CaO:1SiO_2_) was prepared by the sol-gel method using tetraethyl orthosilicate (TEOS, 98% Si (OC_2_H_5_)_4_, Aldrich) and calcium nitrate tetrahydrate (Ca (NO_3_)_2_.4H_2_O, Merck). The mixture of acetic acid and distilled water was used as a catalyst to obtain a homogeneous solution. The synthesis was carried out by hydrolysis and polycondensation of the precursors: TEOS and Ca (NO_3_)_2_.4H_2_O. Acetic acid, nitric acid, or ammonia has been used to catalyze the TEOS hydrolysis during the gelation process and inhibit the formation of crystalline solids [29]. The solutions were rapidly mixed and agitated by a magnetic stirrer for 5 h and then kept at 60 °C for overnight to let the gel formation. After 2 days, the produced translucent gel became dried at 120 °C. Both aging and drying of the wet gels had been carried out at 80 °C after several days on the heating of 5 °C min^−1^ to eliminate residual nitrates. After drying, the sol-gel derived powder was milled in a high-speed porcelain mill to obtain a white powder. In order to get a hard material, the prepared calcium silicate powder became sintered at 1000 °C according to the thermogravimetric analysis.

#### Preparation of nanocomposites materials

The nanocomposites were prepared via mixing nano-HA with calcium silicate and TiO_2_ nanopowders in different ratios as shown in Table 2. For each nanocomposite, HA, calcium silicate, and TiO_2_ nanopowders were thoroughly mixed using alcohol and then dried at 80 °C. The powder samples were uniaxially pressed at a pressure of 1200 MPa to form discs and then fired at 1000 °C for 2 h.

**Table 2:** Composition (weight %) of the prepared ceramic nanocomposites

Sample	HA	TiO2	CS
**C1**	60	20	20
**C2**	60	10	30
**C3**	60	30	10

#### Characterization

##### X-ray diffraction (XRD) of the prepared nanocomposites

XRD analysis was achieved to investigate the phase composition and crystallinity of the sample. The functional properties of the sample were obtained with a Diano X-ray diffractometer using Cu Ka radiation (*λ* = 0.1542 Å) generated by applying a tube voltage of 40 kV and a tube current of 40 mA. Measurements were performed in the range of 2–70° with a scan speed of 4°/min.

##### Fourier transform infrared spectroscopy (FTIR) of the prepared nanocomposites

To investigate the functional groups of the prepared nanocomposites, FTIR measurements were implemented. KBr discs of 0.5 cm diameter were prepared after mixing with the tested samples (mixing ratio 1:100 sample: KBr) in the mortar and ground into a fine powder. Perkin Elmer Spectrum 2000 FTIR spectrometer Demonstrate 1600, Perkin-Elmer (USA), was utilized to record FTIR spectra at a resolution of 4 cm^−1^.

##### Scanning electron microscopy (SEM) of the prepared nanocomposites

SEM was utilized to study the sample surfaces, alongside energy-dispersive X-ray spectrometry (EDS) for evaluating the sample surface. SEM analysis was attempted utilizing a Jeol JXA-840A, Electron Probe miniaturized scale analyzer (Japan), at 15 kV. Samples were rendered electrically conductive before the examination through gold-sputter covering (SPI-Module Sputter Coater; SPI Supplies, West Chester, Pennsylvania, USA) and were appended to the SEM stub utilizing glue carbon tape. In addition, the surface of the prepared nanocomposites was investigated in vitro analysis with stem cells and in vivo studies to determine the bone formation ability of the investigated nanocomposites.

##### Compressive strength

Nanocomposites were examined to detect the effect of different ratios of the three main constituents (HA, CaSiO_3,_ and TiO_2_) on the mechanical properties of the prepared nanocomposites. Compressive strength was measured using a universal testing machine, Zwick Z010, Germany. The average of the tests was taken for the reliability of the results of the three samples. The samples were cylindrical with dimensions (1 cm × 2 cm), the load cell was 10 kN, and the crosshead speed was 10 mm/min.

### Histological study

#### Subject and methods

Bone samples were fixed with 10% buffered formalin and decalcified with 0.5 M ethylenediaminetetraacetic acid, pH 8.3 (Sigma Aldrich, St. Louis, Missouri, USA). The parietal bone was rinsed with PBS, dehydrated with stepwise ethanol, and embedded in paraffin. Cross sections (5–6 μm) were deparaffinized with xylene, hydrated with a series of graded ethanol, and stained with hematoxylin/eosin (H&E). An optical microscope (CX41, Olympus, Tokyo, Japan) was used to take images of the stained samples and confirm the formation of mineral and calcium deposits [30].

## Results

### The in vitro study

#### Proliferative potential and morphological criteria of human dental pulp-derived mesenchymal stem cells

**Fig. 1:**
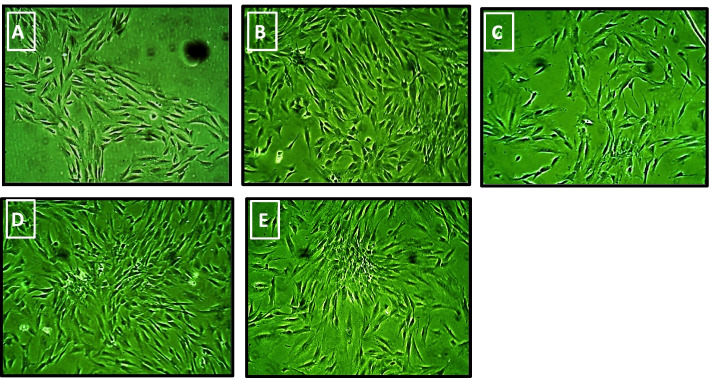
The proliferation and the morphological characteristics of hDP-MSCs derived from a 32-year-old donor during the expansion phase: hDPMSCs were cultured at different passages. (**A**) Primary culture (P0) on day 7. (**B**) and (**C**) First passage (P1) on days 3 and 7, respectively. (**D**) Second passage (P2) on day 4. (**E**) Third passage (P3) on day 5 × 100 magnifications

Human mesenchymal stem cells were isolated from the dental pulp (hDP-MSCs) and cultured in regular proliferation media. The morphological and proliferation characteristics of hDP-MSCs derived from a 32-year-old participant are shown in Fig. 1A–F. The DP-MSCs count in the initial culture was 100 × 10 cells.

Cells appeared after a few days and colonized in the culture plates (P0) (Fig. 1A). After 4 weeks, these colonies formed a confluent monolayer of typical elongated fibroblast-like cells growing into the first passage (P1) (Fig. 1B and C). The morphology of typical spindle mesenchymal stem cells appeared obviously in the subsequent passages as well as their promoted growth forming a condensed monolayer of cells at the second and third passages (P2 and P3) (Fig. 1D and E). This acknowledges the high proliferative capacity of hDP-MSCs.

#### Osteogenic differentiation potential of dental pulp-derived mesenchymal stem cells

Characterization of the osteogenic differentiation of hDP-MSCs was performed by Alizarin Red staining assay to detect the mineralization and calcium deposits produced in the culture.

**Fig. 2:**
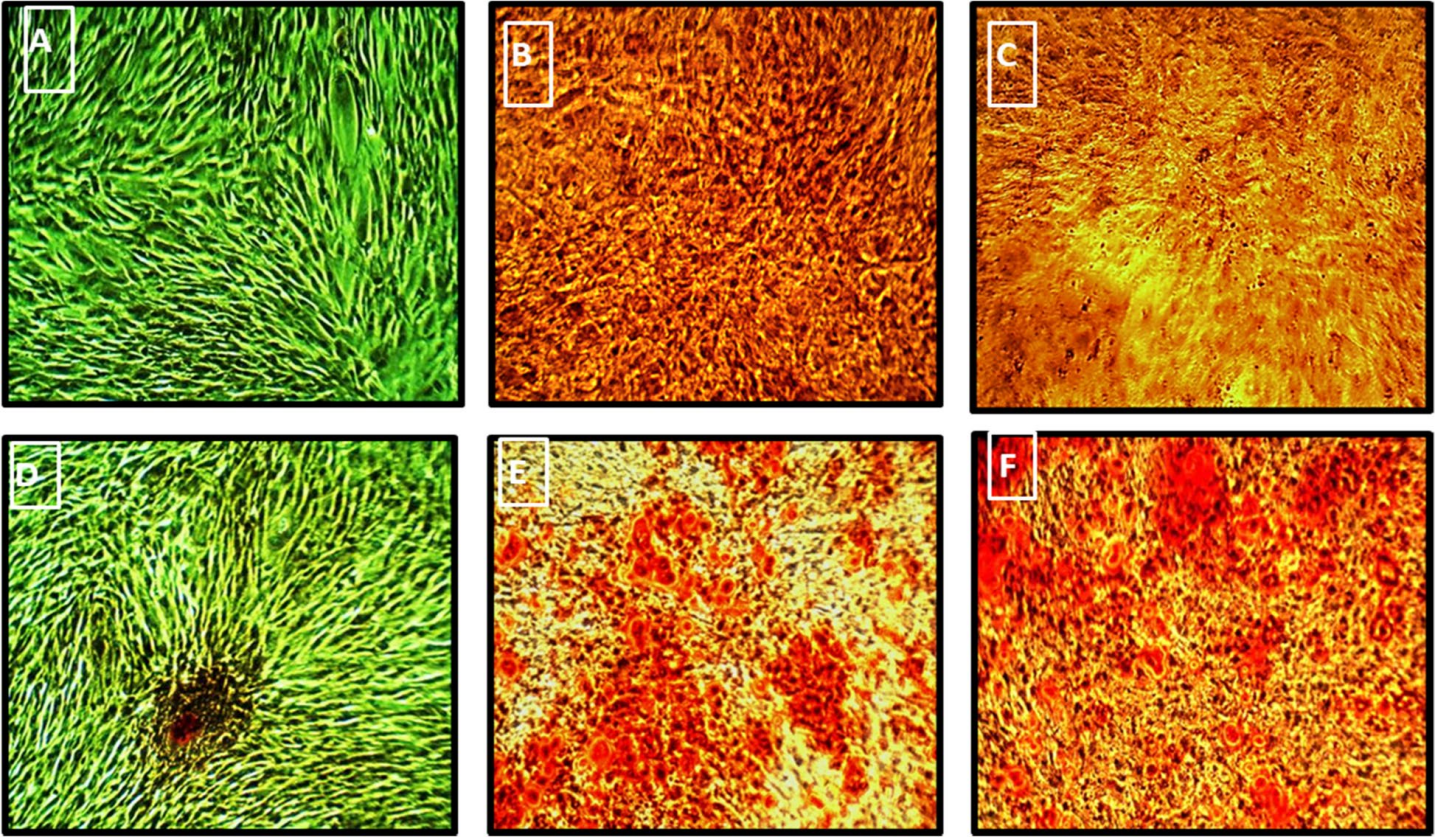
Characterization of the osteogenically differentiated DP-MSCs in the osteogenic differentiated media by Alizarin Red staining on the 14 and 28 days. Unstained control DP-MSCs on the 14th day (**A**). Control showed faint orange stained spot at 28th day (**D**). Represented photos for staining in the osteogenic field on the 14th day (**B**). Moderate Alizarin staining in between the induced cells (**C**). Strong staining pattern in peripheries (**E**). Strong Alizarin staining on the 28^th^ day, in the osteogenic set in between the induced cells. (**F**) Strong Alizarin staining in the periphery of the osteogenic plate. Magnification power ×100

Alizarin stain chelates the minerals and calcium deposits producing an Alizarin Red calcium complex seen as orange-red staining spots (Fig. 2). There were no staining spots in the control plates incubated in regular proliferation media at day 14 (Fig. 2A); however, the control plate presented a faint orange spot on day 28 (Fig. 2D).

By the 14^th^ day after induction of osteogenic differentiation, Alizarin staining revealed moderate stain in the center of the osteogenic plate and a strong one in the periphery (Fig. 2 B and C, respectively). Alizarin Red staining shown on day 28 of the osteogenic differentiation cultures highlighted the increase in the mineralized matrix produced by the differentiated cells.

Several stained spots were observed centrally and peripherally all over the osteogenic plate (Fig. 2E and F, respectively).

#### Flow cytometric markers’ quantification

**Fig. 3:**
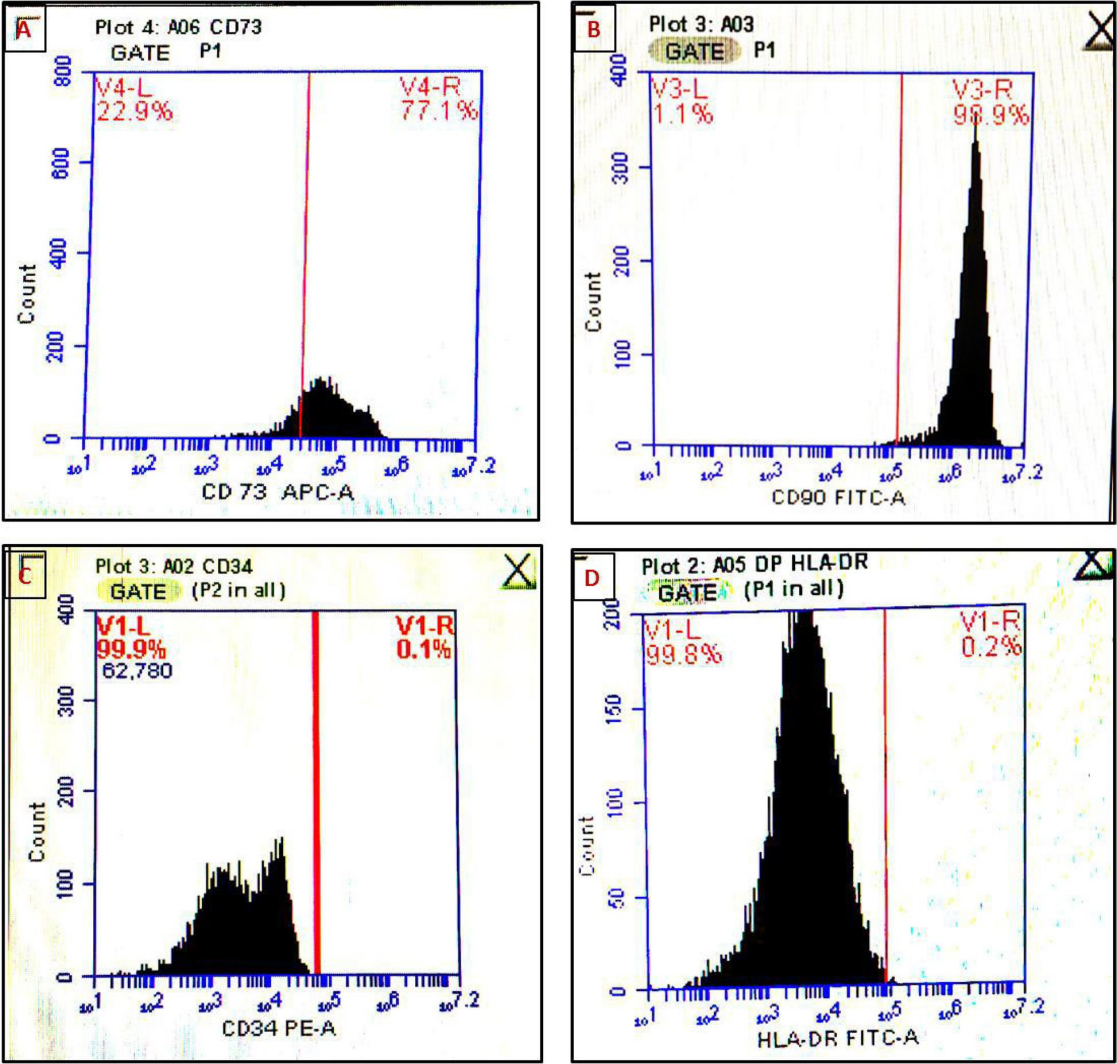
Flow cytometric examinations of hDP-MSCs. **A** and **B** hDP-MSCs were 77.0% and 98.9% positive for both CD73 and CD90 cell surface markers, respectively. **C** and **D** hDP-MSCs were 0.1% and 0.2% negative for both CD34 and HLA-DR cell surface markers, respectively

The DP-MSCs immunophenotypes surface markers measured by the flow cytometry showed a positive rate of 77.0% and 98.9% for the cell surface markers CD73 and CD90, respectively, while the negative rates for the cell surface markers CD34 and HLA-DR were 0.1% and 0.2%, respectively (Table 3) (Fig. 3).

**Table 3:** Flow cytometry of the hDP-MSCs immunophenotypes surface markers

hDP-MSCs immunophenotypes surface markers	Percentage rate %
CD 73	77.0
CD 90	98.9
CD34	0.1
HLA-DR	0.2

### Biomaterial results

#### Phase analysis (XRD)

**Fig. 4:**
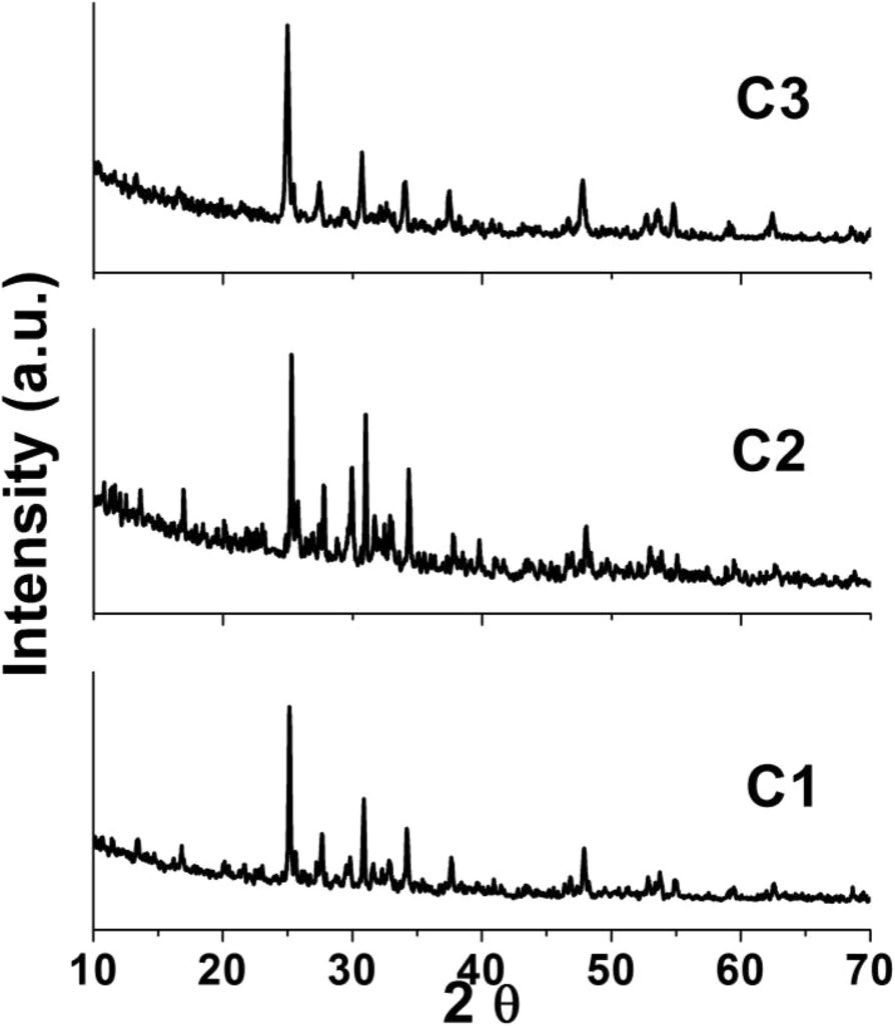
XRD patterns of the prepared ceramic nanocomposite

The XRD patterns of the prepared ceramic nanocomposites (C_1_, C_2,_ and C_3_) fired at 1000 °C are represented in Fig. 4a, b, and c, respectively. All ceramic nanocomposites revealed a well crystalline structure. The patterns revealed the presence of β-tricalcium phosphates (β-TCP) at *dÅ* = 8.15, 3.21, 2.61, and 2.88 in all samples as the main phase along with titania as the second one at *d* = 3.16, 2.83, 2.62, and 5.04 Å and β-CaSiO_3_ (β-wollastonite) (JCPD 84-0654) [28]. Minor lines of hydroxyapatite structure for all ceramic nanocomposites at *dÅ* = 2.81, 2.78, 2.72, and 8.17 are recorded. We noted that the C_3_ sample with higher content of TiO_2_ enhanced the hydroxyapatite (HA) transformation to β-tricalcium phosphate (β-TCP). There are some lines of tetracalcium phosphate (TTCP) at *dÅ* = 2.83, 1.94, and 1.89 (JCPDS card no. 09-0348) in sample C_3_ [21, 31].

#### FTIR

**Fig. 5:**
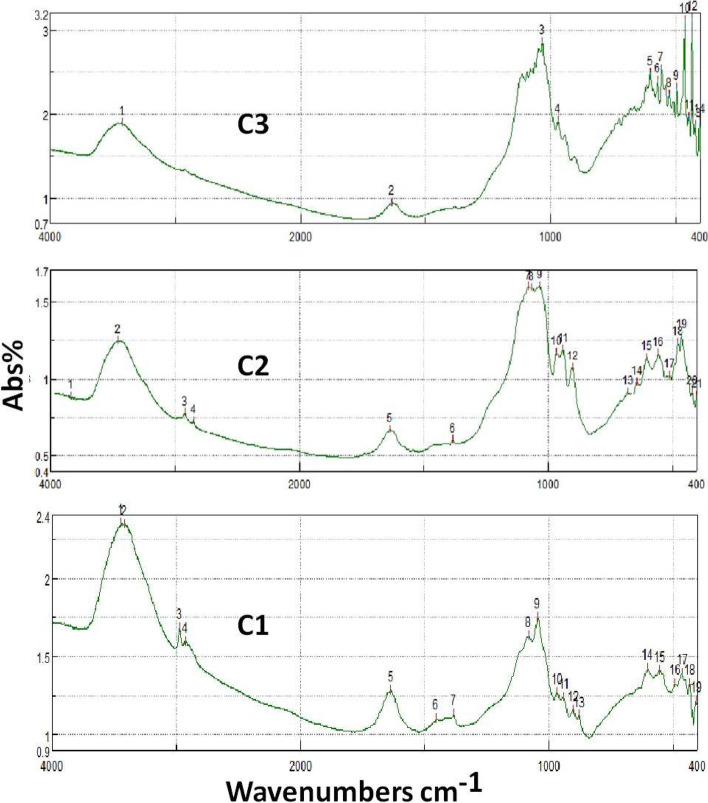
FTIR spectra of the prepared ceramic nanocomposite

The FTIR spectra of the prepared ceramic nanocomposites (C_1_, C_2_, and C_3_) have been recorded by FTIR spectrometer with a 5 cm^−1^ resolution. FTIR spectrum has been recorded at 4000–400 cm^−1^. The FTIR spectra of the prepared ceramic nanocomposites are demonstrated in Fig. 5a, b, and c, respectively. The peaks were observed for all samples at 3391, 1557, and 880 cm^−1^ were due to the OH stretching and bending vibrations, respectively. The peak observed at 1165 cm^−1^ corresponds to Si-O-Si antisymmetric stretching vibration. The peak obtained for all samples at 923 cm^−1^ corresponds to Si-O-Ti vibration. The peak observed for all samples at 801 cm^−1^ is due to the Si-O-Si group.

Three bands were observed as a confirmation for the presence of TiO_2_. Firstly, the band detected at 3500 cm^−1^ is the broadest one and corresponds to the stretching vibration of the hydroxyl group O-H of the TiO_2_. Secondly, bending modes of water Ti-OH were detected around 1630 cm^−1^. Ti-O modes were also detected at 1383 cm^−1^ as the third band that confirms the presence of TiO_2_ [21, 23]. For samples C_1_ and C_2_, the presence of β-TCP was confirmed by the detection of bands in the range of 400–860cm^−1^ that corresponds to the bending phosphate groups. Moreover, detection of bands at 950–1100cm^−1^ related to the stretching phosphate groups was observed. For sample C_3_, both characteristic bans related to β-TCP and TTCP were noted.

#### Mechanical properties

**Fig. 6:**
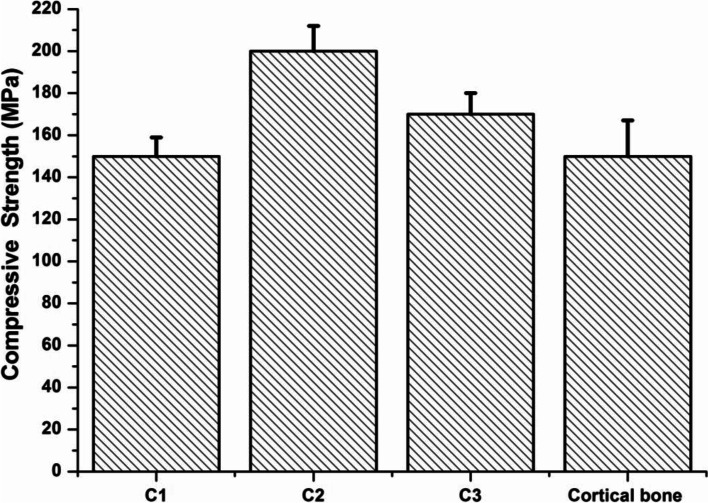
Compressive strength of the prepared ceramic nanocomposites with reference to cortical bone

The results of the compressive strength of the prepared ceramic nanocomposites were illustrated in Fig. 6. Results showed a remarkable increase in the compressive strength of the composite with the highest content of calcium silicate in C2 to 190 MPa. For the C1 ceramic nanocomposite, the strength value declined to reach 150 MPa due to the intrinsic brittleness of the β-TCP with TTCP structure.

#### SEM

**Fig. 7:**
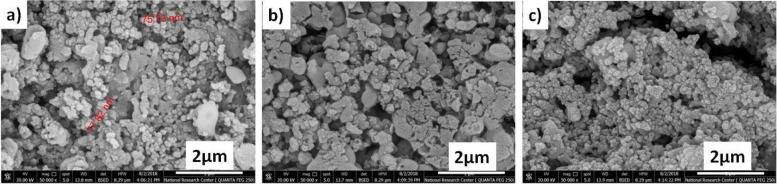
SEM images of the ceramic nanocomposite. **a** C1, **b** C2, and **c** C3

The SEM micrographs of the surface of the β-TCP samples presented in Fig. 7 revealed compact and uniform granular fine structure. The mean grain sizes were in nanoscale for all samples even after higher temperature firing (1000 °C). The mean grain size of the sample C_1_ (Fig. 7(a)) sintered at 1000 °C was in the form of 75–85 nm. Samples C_2_ and C_3_ demonstrated aggregates of nanoparticles of β-TCP with TiO_2_ as shown in Fig. 7c and d. The SEM images of the surfaces demonstrated great similarity in the morphology of the particles when comparing the three samples. The gaps seen between grains suggested that grain boundaries dissolved faster compared to the core of the grains. The TCP structure is highly agglomerated with almost spherical particles having an average size of 65 nm. The necking among the particles was apparent and thus could be due to localized high heat treatment (1000 °C). The surfaces of all the prepared samples were highly agglomerated due to the formation of beta-tricalcium phosphate along with the presence of CS and TiO_2_; they consist of smaller particles, 72–85 nm.

#### SEM after cells seeded

**Fig. 8:**
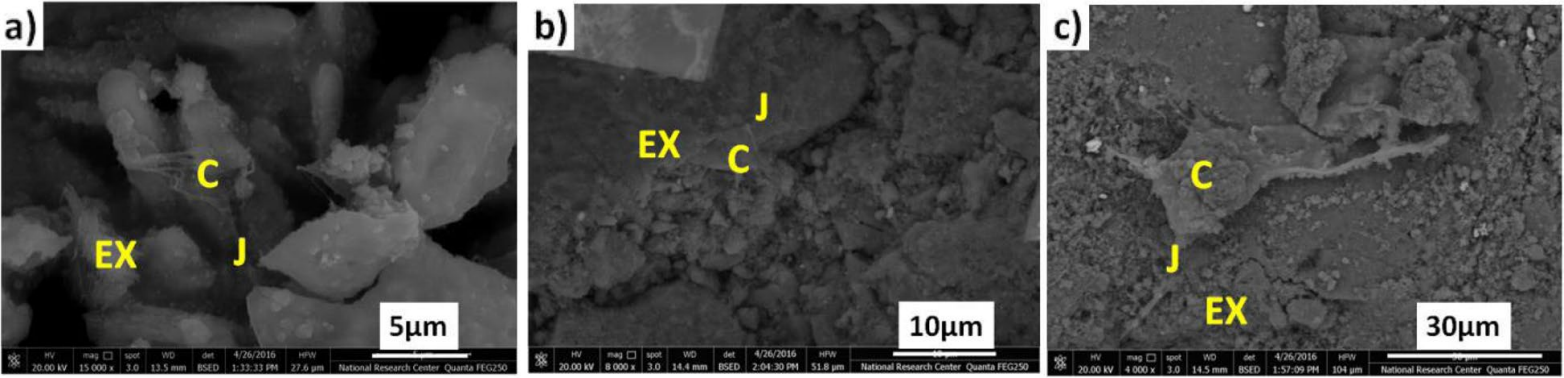
SEM images for cells attachment on the surface of ceramic nanocomposite. **a** C1, **b** C2, and **c** C3

The hDP-MSCs were seeded on the prepared ceramic nanocomposites surface, showed an elongated form, and extended out discretely inside the pores and at the surface of all the prepared samples. Furthermore, after 3 weeks of culture, the formation of mineral aggregates was observed, demonstrating the osteogenic differentiation of all the prepared samples. The higher magnification images (Fig. 8a, b, and c) for samples C1, C2, and C3, respectively. These SEM micrographs exhibited rough structures (J) that confirm the presence of cells (C) on all the prepared samples, as well as indicating greater extracellular matrix (EX) deposition and fibrous networks on all the prepared samples surfaces accompanied with cells attachment.

### In vivo study

#### Evaluation of rabbit tibial defect’s healing after transplantation

**Fig. 9:**
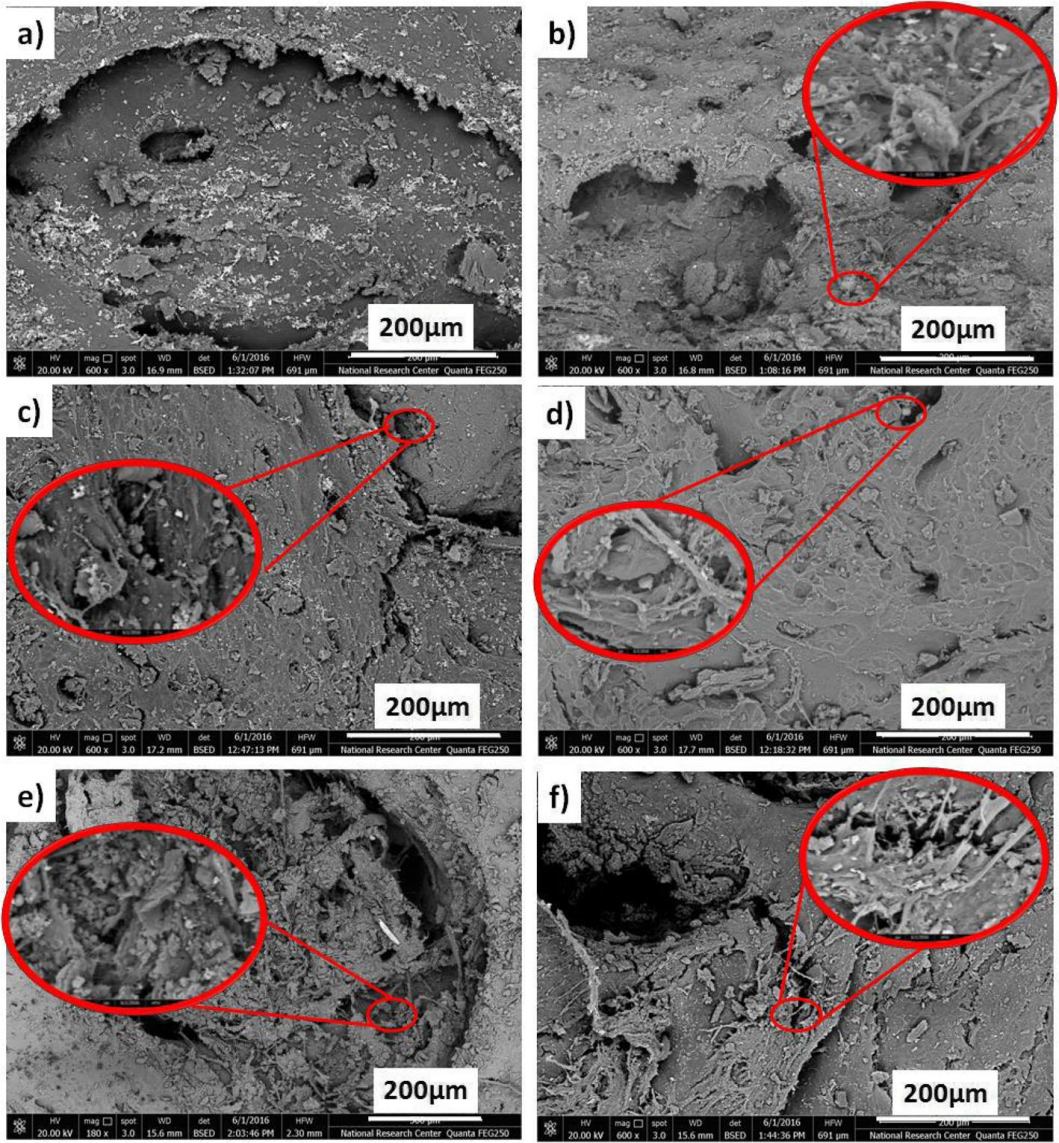
SEM images for post-implantation ceramic nanocomposite. C1, **a** and **b**; C2, **c** and **d**; and C3, **e** and **f**

Rabbits were sacrificed on the 4^th^ week following the implantation. Electron microscope and histopathological analysis were performed. Bone healing was examined in vivo using SEM (Fig. 9a, b, c) for samples C1, C2, and C3, respectively. There was no abnormality detected at the surgical site of the rabbit tibial defects, indicating normal healing of bone tissue. All rabbits were healthy without presenting any indications of edema through the post-transplantation period. The originally prepared ceramic nanocomposites interacted directly with the new bone without any fibrous encapsulation and were almost completely filled with new bones (NB), making the prepared ceramic nanocomposites highly bioactive and biocompatible.

Some debris and unfilled edges of the prepared ceramic nanocomposites were still found in the rabbit tibial defects. They are in direct contact with new bone without any fibrous encapsulation and are filled with bone. This indicates that the prepared ceramic nanocomposites are highly bioactive and biocompatible. Bone defects induced in the tibia of rabbits 4 weeks after transplantation of unseeded ceramic nanocomposites were semi-healed with a highly mineralized bone matrix and kept the tibial defect areas and were semi-opened (Fig. 9a, c, e). The rabbit tibial defects were transplanted with osteogenically differentiated hDP-MSCs, 4 week post-transplantation (Fig. 9b, d, f), the newly formed bone proceeded inward from the deep end wall of the tibial defect areas. The complete bone incorporation was demonstrated for the osteogenically differentiated hDP-MSCs with the prepared ceramic nanocomposites 4 weeks after transplantation (Fig. 9c). New bones were continuously formed from the surface of the graft to the cortical bone at the defect site to the bone marrow area. In addition, SEM results performed on the unseeded ceramic nanocomposites transplanted in vivo showed significant reaction with the host bone, and the remodeling progressed at the ceramic-nanocomposites-bone boundary. The experimental group of rabbit tibial defect 4 weeks after implantation was completely filled with calcified (M) bone matrix, collagen fibers (CF), and differentiated dental pulp stem cells (DC) (Fig. 9a, c, d). There was an indication of the newly formed bone on the surface of all transplants, but no sign of fibrous encapsulation with symmetrical stretch mode.

### Histopathological results

#### Dental pulp stem cell with ceramic nanocomposites scaffold

**Fig. 10:**
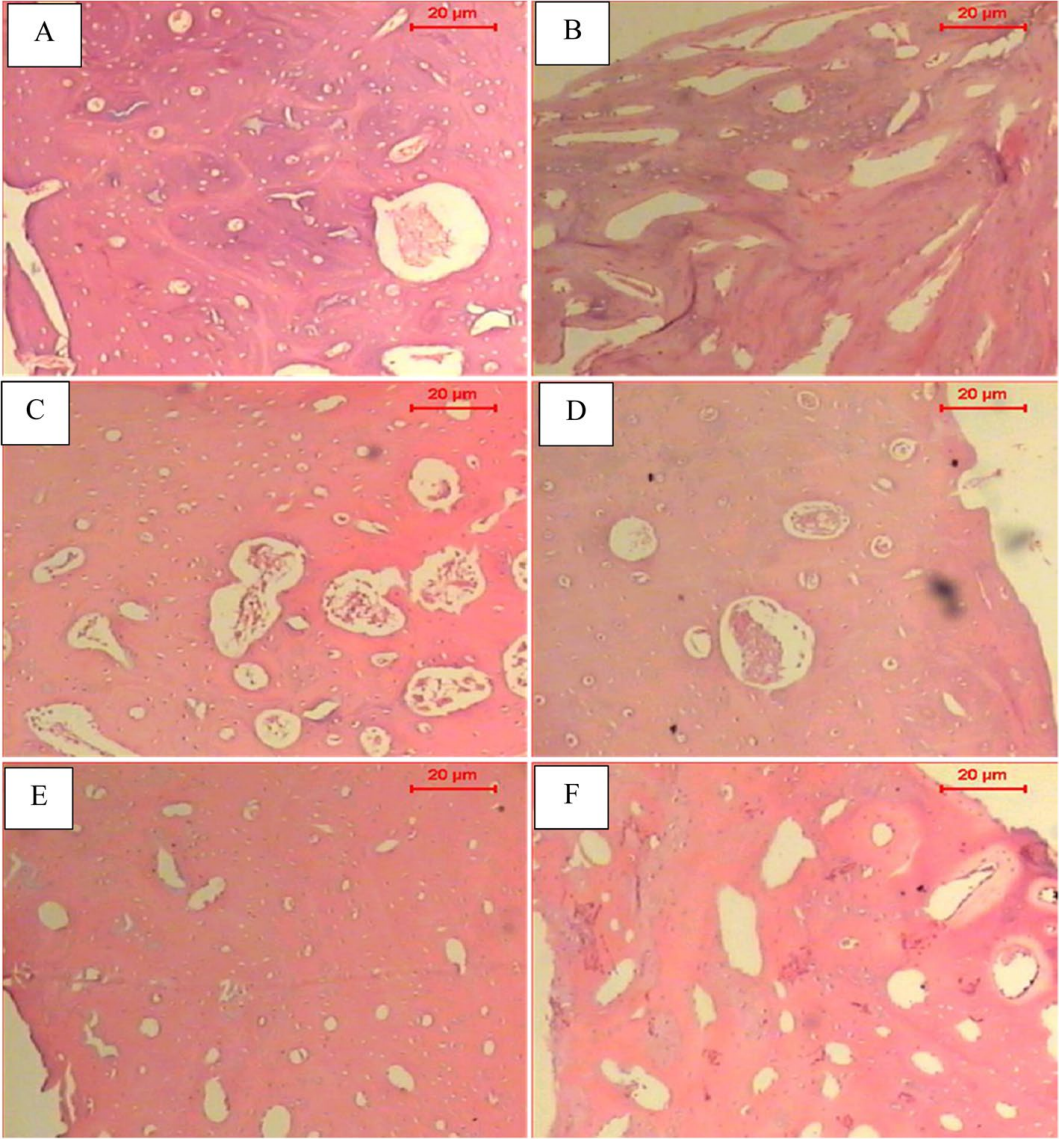
Histopathological sections of rabbits transplanted by hDP-MSCs and ceramic nanocomposites scaffold. Sections of bone of **A** tested dental pulp stem cells (DP-MSCs) with ceramic nanocomposites (C1) after 4 weeks show nodules like shape of new formed bone and few vacuoles that are filled with connective tissue. **B** Control of ceramic nanocomposites (C1) only after 4 weeks shows many of vacuoles surrounded by new formed bone. **C** Tested dental pulp stem cells (DP-MSCs) with ceramic nanocomposites (C2). After 4 weeks, extensive regions of the mature bone showed in the defect parts. Note the collagen-filled vacuole. **D** Control of ceramic nanocomposites (C2) only after 4 weeks shows thickness of bone regeneration in the defect. Few vacuoles that are filled with connective tissues. **E** Tested dental pulp stem cells (DP-MSCs) with ceramic nanocomposites (C3) after 4 weeks show that the defect is filled with mature bone, and small vacuoles are present. **F** Control of ceramic nanocomposites (C3) after 4 weeks large vacuoles embedded in the new formed bone (H&E stain; scale bar: 20 μm)

Sections of bone of the dental pulp stem cells (DP-MSCs) seeded on ceramic nanocomposites (C1) scaffold 4 weeks post-transplantation showed nodules like the shape of new-formed bone and few vacuoles that filled with connective tissue (Fig. 10A). The histological analysis of the control, ceramic nanocomposites (C1) scaffold, showed many vacuoles surrounded by newly formed bone (Fig. 10B).

Microscopic examination of the dental pulp stem cells (DP-MSCs) seeded on ceramic nanocomposites (C2) scaffold 4 weeks post-transplantation presented extensive regions of the mature bone shown in the defect parts. Moreover, some collagen-filled vacuoles were observed (Fig. 10C). In addition, the examination of the control, ceramic nanocomposites (C2) scaffold, 4 weeks post-transplantation showed the thickness of bone regeneration in the defect site; besides, few vacuoles that filled with connective tissues were shown (Fig. 10D).

The histological analysis of bone defect sections having the dental pulp stem cells (DP-MSCs) seeded on ceramic nanocomposites (C3) 4 weeks post-transplantation showed the defect area well-filled with mature bone; small vacuoles were detected (Fig. 10E). However, in the control, ceramic nanocomposites (C3) scaffold, only large vacuoles were seen embedded in the newly formed bone (Fig. 10F).

## Discussion

The dental pulp (DP) is an auspicious and easily accessible source of MSCs for bone tissue engineering. Human dental pulp stem cells (hDP-MSCs) isolated from the third molar play a vital role in regenerative medicine. Several studies have considered the significance of hDP-MSCs in bone tissue engineering. However, the types of scaffolds used for enhancement of the hDP-MSCs proliferation as well as its osteogenic differentiation, and applications in animal models with induced bone defects, were highly diverse [1–5].

hDP-MSCs have been investigated by our group as a cell source for craniofacial bone regeneration in rabbit models [13]. In the present study, the combination of hDP-MSCs and ceramic nanocomposites system has been suggested as an approach for bone tissue engineering in a rabbit model with induced tibial defects. Our findings revealed the role of the combined application of hDP-MSCs and the originally prepared ceramic nanocomposites scaffold (C1, C2, C3) in enhancing the osteogenic induction of hDP-MSCs seeded over the modular ceramic nanocomposites for 3 weeks in vitro culture. The in vivo results showed the extensive calcification produced and repair of bone defects induced in the rabbit models.

The DP-MSCs osteogenic differentiation efficiency has been investigated in several studies [32–35]. Alizarin Red staining was used to assess the osteoblastic phenotype for calcium deposition; Alizarin staining chelates the calcium nodules, producing orange-red spots. The strong intensity staining pattern in our in vitro cultures indicated the occurrence of osteogenic differentiation on days 14 and 28 post the initial osteogenic induction cultures.

In this study, Alizarin Red staining was used to assess the mineralization and spread of the mineralized nodules produced by the hDP-MSCs seeded on ceramic nanocomposite discs (C1, C2, and C3). Our findings demonstrated the remarkable increase in cultures’ mineralization when using a combination of hDP-MSCs and ceramic nanocomposite system 3 weeks post the in vitro culture experiments.

The flow cytometry analysis was performed here as an additional confirmatory evidence of the phenotypic characteristics of hDP-MSCs stemness by detecting the expression percentage of surface markers. Our results showed moderate expression of CD73 (77.0%) as well as high expression of CD90 (98.9%) while rather a negative expression of CD34 and HLA-DR (0.1% and 0.2%, respectively). The unique hDP-MSCs surface markers expression pattern has been confirmed in several other studies [34, 36–44]. A controversy with regard to the expression of surface marker CD34 was reported in few other studies [10, 34].

Extensive research in the era of bone tissue engineering and bone repair has been carried out aiming at exploring the potential of the combined use of osteogenic-differentiated hDP-MSCs together with different preparations of scaffold materials. Alipour et al. (2021) has used a certain type of scaffold, the alginate-gelatin microcapsules containing nHA in combination with hDPSCs. Their results revealed that the microencapsulation of hDPSCs in the Alg/Gel/nHA hydrogel could be a prospective approach for dentistry regeneration [35]. The formation of a bone-like hard tissue following the subcutaneous implantation of differentiated rat-derived DP-MSCs seeded on a three-dimensional porous calcium phosphate ceramic scaffold in an immunocompromised mouse has been demonstrated [32].

It has been found that the compressive strength of the synthesized ceramic nanocomposites is enhanced by the addition of calcium silicate to the hydroxyapatite [21]. The results shown here demonstrated that the increase of the calcium silicate content led to an increase in the strength value of the ceramic nanocomposites. The compressive strength values of reported ceramic nanocomposites exceed the strength values of human cortical bone (100–230 MPa) [31]. Previous studies have recorded a compressive strength of 150 MPa for the pure hydroxyapatite (HA) [45]. As the compressive strength of nanocomposite increases, it will be more similar to the natural bones and has a greater potential for application in bone repair. The prepared ceramic nanocomposite, C2, displays the highest compressive strength value, 190 Mpa.

In the present study, the nanocomposites were fabricated by mixing nano-hydroxyapatite (nano-HA) with calcium silicate and TiO_2_ nanopowders in different ratios (C1, C2, and C3). These nanocomposites, particularly the C2, showed the highest enhancement effect in the osteogenic differentiation of hDP-MSCs both in in vitro cultures and in vivo animals model, the experimentally induced tibial defect.

Different types of synthesized nanocomposites have shown a role in dentin regeneration and bone repair. In Corral Nunez et al. (2022) study, a novel nanocomposite containing bioactive glass nanoparticles (nBGs) and Biodentine (BD) (nBG/BD) has led to a faster dentin formation and is suggested to be a promising material in healing and repair of the dentin-pulp complex [46]. In another study, originally prepared magnetic nanoparticles that contain iron oxide and Mg-phosphate ceramic (nMgP-Fe) and impregnated with DP-MSCs were shown to play a role in bone disease therapy and dental pulp regeneration [47].

In a previous study, we demonstrated the combination of 3D scaffold that contains 30% nano-hydroxyapatite chitosan together with the 2^nd^ trimester human amniotic fluid stem cells (hAF-MSCs) was shown to have a therapeutic potential in the healing of an induced rabbit tibial defects [48].

Interestingly, the SEM micrographs of the originally prepared nanocomposites for the present study exhibited rough structures that confirm the presence of cells on all the prepared composite’s samples and showed dense deposits of extracellular matrix and fibrous networks on the surface of all the prepared composites that accompanied the attached cells.

The histopathological analysis of our experimental and control rabbit groups revealed obvious differences in the process of bone formation. The results demonstrated enhancement of bone healing in the experimental rabbit groups with the induced tibial defects transplanted with the combined application of osteogenically differentiated hDP-MSCs cells and ceramic nanocomposites. The defect was filled, mainly with mature bone as well as some collagen-filled follicles 4 weeks post-implantation. Nevertheless, the control rabbit groups with induced tibial defects transplanted with only ceramic nanocomposite scaffolds (C1, C2, C3) showed less calcified tissue, restricted newly formed bone, and large areas of the bone defect that were not yet filled with new bone formation.

The histology outcome confirms the superior transplantation effect of the combined application of our novel prepared composites C1, C2, and C3, impregnated with hDP-MSCs in producing efficient bone formation and repair at the tibial site of the induced defect.

In a rather similar approach study but of different scaffold and animal models, Kwon et al. (2015) have used the hDP-MSCs seeded on a computer-designed scaffold made from biodegradable polyesters and solid free-form fabrication, to investigate the bone repair of an induced cranial defect in a rat model. Huge bone-like ingrowths, in comparison with the control group, were gradually observed at three-time points of 4, 8, and 12 weeks [49].

## Conclusion

Here, we have demonstrated the efficacy of the combined application of originally prepared nanocomposites impregnated with hDP-MSCs in promoting osteogenic differentiation as well as new bone formation at a critical-sized induced bone defect in a rabbit model. The novel prepared ceramic nanocomposites have been shown to promote the deposition of collagen, calcium, and phosphate, enhancing the new bone formation 4 weeks post-implantation. Our novel ceramic nanocomposite is recommended for further applications in bone tissue engineering and regenerative medicine.

## Data Availability

All data generated or analyzed during this study are included in this published article.

## Abbreviations

FACS: Fluorescein-activated cell sorting
hDP-MSCs: Human dental pulp mesenchymal stem cells
hNDPSCs: Human natal dental pulp stem cells
nHA: Nano-hydroxyapatite
SEM: Scanning electron microscopy
TMJ: Temporomandibular joint
XRD: X-ray diffraction

## References

[1] Leyendecker Junior A, Gomes Pinheiro CC, Lazzaretti Fernandes T, Franco Bueno D (2018). The use of human dental pulp stem cells for in vivo bone tissue engineering: a systematic review. J Tissue Eng.

[2] Akiyama K, Chen C, Gronthos S, Shi S (2012). Lineage differentiation of mesenchymal stem cells from dental pulp, apical papilla, and periodontal ligament. Methods Mol Biol.

[3] Gronthos S, Brahim J, Li W, Fisher LW, Cherman N, Boyde A, DenBesten P, Gehron Robey P, Shi S (2002). Stem cell properties of human dental pulp stem cells. J Dent Res.

[4] Graziano A, d’Aquino R, Laino G, Papaccio G (2008). Dental pulp stem cells: a promising tool for bone regeneration. Stem Cell Rev.

[5] Undale AH, Westendorf JJ, Yaszemski MJ, Khosla S (2009). Mesenchymal stem cells for bone repair and metabolic bone diseases. Mayo Clin Proc.

[6] Kern S, Eichler H, Stoeve J, Klüter H, Bieback K (2006). Comparative analysis of mesenchymal stem cells from bone marrow, umbilical cord blood, or adipose tissue. Stem Cells.

[7] Jaquiéry C, Schaeren S, Farhadi J, Mainil-Varlet P, Kunz C, Zeilhofer HF, Heberer M, Martin I (2005). In vitro osteogenic differentiation and in vivo bone-forming capacity of human isogenic jaw periosteal cells and bone marrow stromal cells. Ann Surg.

[8] Uccelli A, Moretta L, Pistoia V (2008). Mesenchymal stem cells in health and disease. Nat Rev Immunol.

[9] Pisciotta A, Carnevale G, Meloni S, Riccio M, De Biasi S, Gibellini L, Ferrari A, Bruzzesi G, De Pol A (2015). Human dental pulp stem cells (hDPSCs): isolation, enrichment and comparative differentiation of two sub-populations. BMC Dev Biol.

[10] Kawashima N (2012). Characterisation of dental pulp stem cells: a new horizon for tissue regeneration?. Arch Oral Biol.

[11] Liu M, Lv Y (2018). Reconstructing bone with natural bone graft: a review of in vivo studies in bone defect animal model. Nanomaterials (Basel).

[12] Che Seman C, Zakaria Z, Sharifudin MA, Che Ahmad A, Ms A, Yusof N, Buyong Z (2018) Model of a critical size defect in the new zealand white rabbit's tibia. Int Med J Malaysia 17(1):13-18

[13] Mohammed EEA, El-Zawahry M, ARH F, Abdel Aziz NN, El-Mohandes WA, Abou-Shahba N, Mahmoud M, El-Farmawy MA, Aleem AK (2019). Osteogenic potential of human dental pulp-derived mesenchymal stem cells in bone regeneration of rabbit. J Arab Soc Med Res.

[14] Chiara G, Letizia F, Lorenzo F, Edoardo S, Diego S, Stefano S, Eriberto B, Barbara Z (2012). Nanostructured biomaterials for tissue engineered bone tissue reconstruction. Int J Mol Sci.

[15] Nievethitha SS, Subhapradha N, Saravanan D, Selvamurugan N, Tsai WB, Srinivasan N, Murugesan R, Moorthi A (2017). Nanoceramics on osteoblast proliferation and differentiation in bone tissue engineering. Int J Biol Macromol.

[16] Giannoudis PV, Dinopoulos H, Tsiridis E (2005). Bone substitutes: an update. Injury.

[17] Wang XH, Zhou YN, Xia LG, Zhao CC, Chen L, Yi DL, Chang J, Huang LP, Zheng XB, Zhu HY (2015). Fabrication of nano-structured calcium silicate coatings with enhanced stability, bioactivity and osteogenic and angiogenic activity. Colloid Surface B.

[18] Gotz W, Tobiasch E, Witzleben S, Schulze M (2019) Effects of silicon compounds on biomineralization, osteogenesis, and hard tissue formation. Pharmaceutics 11(3):117 10.3390/pharmaceutics11030117 PMC6471146 30871062

[19] Roohani-Esfahani SI, No YJ, Lu ZF, Ng PY, Chen YJ, Shi J, Pavlos NJ, Zreiqat H (2016) A bioceramic with enhanced osteogenic properties to regulate the function of osteoblastic and osteocalastic cells for bone tissue regeneration. Biomed Mater 11(3):035018 10.1088/1748-6041/11/3/035018 27305523

[20] Manchon A, Alkhraisat M, Rueda-Rodriguez C, Torres J, Prados-Frutos JC, Ewald A, Gbureck U, Azama JC, Rodriguez-Gonzalez A, Lopez-Cabarcos E (2015). Silicon calcium phosphate ceramic as novel biomaterial to simulate the bone regenerative properties of autologous bone. J Biomed Mater Res A.

[21] Beheri HH, Mohamed KR, El-Bassyouni GT (2013). Mechanical and microstructure of reinforced hydroxyapatite/calcium silicate nano-composites materials. Mater Design.

[22] Singh Z (2018). Nanoceramics in bone tissue engineering: the future lies ahead. Trends J Sci Res.

[23] Beherei HH, Mohamed KR, El-Bassyouni GT (2009). Fabrication and characterization of bioactive glass (45S5)/titania biocomposites. Ceram Int.

[24] Miura M, Gronthos S, Zhao M, Lu B, Fisher LW, Robey PG, Shi S (2003). SHED: stem cells from human exfoliated deciduous teeth. Proc Natl Acad Sci U S A.

[25] Zhang Q, Wang X, Chen Z, Liu G (2007). Semi-quantitative RT-PCR analysis of LIM mineralization protein 1 and its associated molecules in cultured human dental pulp cells. Arch Oral Biol.

[26] Tsukamoto Y, Fukutani S, Shin-Ike T, Kubota T, Sato S, Suzuki Y, M. (1992). M: Mineralized nodule formation by cultures of human dental pulp-derived fibroblasts. Arch Oral Biol.

[27] RH. J: Flow cytometry protocols. Eds Humana Press, Totowa, 1998:217-238.

[28] Matsiko A, Gleeson JP, O'Brien FJ (2015). Scaffold mean pore size influences mesenchymal stem cell chondrogenic differentiation and matrix deposition. Tissue Eng Pt A.

[29] Hung IM, Shih WJ, Hon MH, Wang MC (2012). The properties of sintered calcium phosphate with [Ca]/[P]=1.50. Int J Mol Sci.

[30] Carleton HM, Drury RAB, Wallington EA (1980) Carleton’s histological technique. New York: Oxford University Press

[31] León A, Reuquen P, Garín C, Segura R, Vargas P, Zapata P, Orihuela PA (2017). FTIR and Raman characterization of TiO2 nanoparticles coated with polyethylene glycol as carrier for 2-methoxyestradiol. Appl Sci.

[32] Karbanova J, Soukup T, Suchanek JMJ (2010). Osteogenic differentiation of human dental pulp-derived stem cells under various ex-vivo culture conditions. Acta Med (Hradec Kralove).

[33] Laino G, d’Aquino R, Graziano A, Lanza V, Carinci F, Naro F, Pirozzi G, Papaccio G (2005). A new population of human adult dental pulp stem cells: a useful source of living autologous fibrous bone tissue (LAB). J Bone Miner Res.

[34] Bakopoulou A, Leyhausen G, Volk J, Tsiftsoglou A, Garefis P, Koidis P, Geurtsen W (2011). Comparative analysis of in vitro osteo/odontogenic differentiation potential of human dental pulp stem cells (DPSCs) and stem cells from the apical papilla (SCAP). Arch Oral Biol.

[35] Alipour M, Firouzi N, Aghazadeh Z, Samiei M, Montazersaheb S, Khoshfetrat AB, Aghazadeh M (2021). The osteogenic differentiation of human dental pulp stem cells in alginate-gelatin/nano-hydroxyapatite microcapsules. BMC Biotechnol.

[36] Karaoz E, Dogan BN, Aksoy A, Gacar G, Akyuz S, Ayhan S, Genc ZS, Yuruker S, Duruksu G, Demircan PC (2010). Isolation and in vitro characterisation of dental pulp stem cells from natal teeth. Histochem Cell Biol.

[37] Akpinar G, Kasap M, Aksoy A, Duruksu G, Gacar G, Karaoz E (2014) Phenotypic and proteomic characteristics of human dental pulp derived mesenchymal stem cells from a natal, an exfoliated deciduous, and an impacted third molar tooth. Stem Cells Int 2014:457059. 10.1155/2014/457059 PMC4212660 25379041

[38] Ledesma-Martinez E, Mendoza-Nunez VM, Santiago-Osorio E (2016) Mesenchymal stem cells derived from dental pulp: a review. Stem Cells Int 2016:4709572 10.1155/2016/4709572 PMC4686712 26779263

[39] Suchánek J, Visek B, Soukup T, El-Din Mohamed SK, Ivancaková R, Mokrỳ J, Aboul-Ezz EH, Omran A (2010). Stem cells from human exfoliated deciduous teeth--isolation, long term cultivation and phenotypical analysis. Acta Medica.

[40] Pivoriunas A, Surovas A, Borutinskaite V, Matuzevicius D, Treigyte G, Savickiene J, Tunaitis V, Aldonyte R, Jarmalaviciute A, Suriakaite K (2010). Proteomic analysis of stromal cells derived from the dental pulp of human exfoliated deciduous teeth. Stem Cells Dev.

[41] Bray AF, Cevallos RR, Gazarian K, Lamas M (2014). Human dental pulp stem cells respond to cues from the rat retina and differentiate to express the retinal neuronal marker rhodopsin. Neuroscience.

[42] Lindemann D, Werle SB, Steffens D, Garcia-Godoy F, Pranke P, Casagrande L (2014). Effects of cryopreservation on the characteristics of dental pulp stem cells of intact deciduous teeth. Arch Oral Biol.

[43] Werle SB, Lindemann D, Steffens D, Demarco FF, de Araujo FB, Pranke P, Casagrande L (2016). Carious deciduous teeth are a potential source for dental pulp stem cells. Clin Oral Invest.

[44] Govindasamy V, Abdullah AN, Ronald VS, Musa S, Ab Aziz ZAC, Zain RB, Totey S, Bhonde RR, Abu Kasim NH (2010). Inherent differential propensity of dental pulp stem cells derived from human deciduous and permanent teeth. J Endodont.

[45] Mohamed KR, Beherei HH, El-Bassyouni GT, El Mahallawy N (2013) Fabrication and mechanical evaluation of hydroxyapatite/oxide nano-composite materials. Mater Sci Eng C (33):4126 10.1016/j.msec.2013.05.059 23910323

[46] Corral Nunez C, Altamirano Gaete D, Maureira M, Martin J, Covarrubias C (2021). Nanoparticles of bioactive glass enhance biodentine bioactivity on dental pulp stem cells. Materials (Basel).

[47] Farag MM, Beherei H, Al-Rashidy ZM, Farag DBE, Salem ZA (2022) Dental pulp stem cell viability and osteogenic potential assessment of new Mg-phosphate magnetic bioceramic nanoparticles. J Materials Res 37:595–607

[48] Mohammed EEA, Beherei HH, El-Zawahry M, Farrag ARH, Kholoussi N, Helwa I, Gaber K, Allam MA, Mabrouk M, Aleem AKA (2019). Combination of human amniotic fluid derived-mesenchymal stem cells and nano-hydroxyapatite scaffold enhances bone regeneration. Open Access Maced J Med Sci.

[49] Yeon Kwon D, Seon Kwon J, Hun Park S, Hun Park J, Hee Jang S, Yun Yin X, Yun J-H, Ho Kim J, Hyun Min B, Hee Lee J (2015). A computer-designed scaffold for bone regeneration within cranial defect using human dental pulp stem cells. Sci Rep-Uk.

